# Establishment of a 3-Dimensional Intestinal Cell Model to Simulate the Intestinal Mucosal Immune System for Food Allergy Investigations

**DOI:** 10.3389/fimmu.2022.853443

**Published:** 2022-03-01

**Authors:** Linglin Fu, Wanglei Lin, Chong Wang, Yanbo Wang

**Affiliations:** Food Safety Key Laboratory of Zhejiang Province, School of Food Science and Biotechnology, Zhejiang Gongshang University, Hangzhou, China

**Keywords:** food allergy, cell model, transwell, Hippo pathway, immunity

## Abstract

Food allergy is a worldwide food safety problem with increasing prevalence. Developing novel approaches for food allergy investigations is the basis for controlling food allergies. In this work, a 3-dimensional (3D) intestinal cell model was established to simulate the intestinal mucosal immune system. Gut epithelial cell line CMT93 was cultured in a transwell insert above dendritic cells (DCs) isolated from mouse spleen and stimulated by egg allergen ovalbumin (OVA), then the conditioned media of DCs was transferred to T cells isolated from mouse spleen. The allergy-related indexes of each cell type were determined by qPCR and flow cytometry. Then the TAZ gene was knocked down in the CMT93 cells and the role of the Hippo pathway in OVA-induced food allergy was investigated. The 3D intestinal cell model showed more significant and more specific allergic responses than conventional cell models and is more convenient to be manipulated than the mouse models. This model is an ideal tool for food allergy investigations and would facilitate studies in the field of intestinal mucosal immunity.

## 1 Introduction

In recent years, an increasing number of people are suffering from food allergies, with about 8% of children and 4% of adults having allergies to one or more foods (1, 2). The main allergic reactions to food are attributed only to a limited variety of proteins, which are considered the main allergens (3). For example, ovalbumin (OVA) accounts for half of egg white protein and is the main allergen of eggs, which often causes allergic reactions especially in children (4, 5). Although various approaches have been applied to control food allergies, the most efficient way is to avoid intaking allergens currently. To develop a more positive and effective way to prevent and treat food allergies, more detailed basic mechanisms of food allergy should be elucidated, and relevant fundamental investigations are urgently needed.

Food allergy is a comprehensive physiological process involving various cell types. In the sensitization stage, when entering the intestine, the food allergens first contact with the intestinal epithelial cells (IEC) and are then recognized by the antigen-presenting cells such as dendritic cells (DCs) in the lamina propria beneath IEC. Then the allergen information was transferred to T cells, guiding the differentiation of allergenic Th2 cells, which transfer the allergenic signal to B cells, resulting in the production of allergen-specific IgE. These IgE binds to the surface of mast cells or basophils, making the body sensitized. In the effect stage, the allergen directly interacts with IgE, leads to mast cells or basophils degranulation, and finally results in allergenic responses (6, 7).

Due to the complexity of food allergy reactions, cells models are usually unable to simulate the process, so animal models and cohort studies are the major approaches for food allergy investigations, especially for mechanism studies. However, compared with cell models, animal models and cohort studies require higher economic and time costs, are uneasy to scale up for high-throughput researches, limit in manipulation possibility, and may cause ethical problems. Therefore, numerous researchers have spent great efforts to establish an ideal cell model for food allergy investigations. Degranulation models are the most commonly used type of food allergy cell models, which mainly use LAD2, RBL-2H3, HMC-1, or other basophils or mast cell lines (8–10). However, these models focus on the effect stage rather than the sensitization stage of food allergy and are widely used for assessing the allergenicity of allergens but not for investigating the mechanisms of allergy reactions. On the other hand, by using the dendritic cells derived from mouse bone marrow, human peripheral blood mononuclear cells, and THP1 cell line, Tsai and colleagues demonstrated how dust mite allergen Der p 7 activates DCs and T cells (11). By using a mouse gut epithelial cell line CMT93, Wang and colleagues elucidated the molecular mechanisms of how shrimp allergen tropomyosin irritates the allergenic potential of IEC (12). Moreover, several studies tried to combine different types of cells to establish a more complex cell model. Sae and colleagues established a transwell co-culture system consisting of human IEC cell line Caco-2 and mast cell line RBL-2H3, which can evaluate the effects of food factors that inhibit mast cell degranulation (13). By using a transwell system to combine IECs and peripheral blood mononuclear cells, Kivit and colleagues revealed the anti-allergenic activity of nondigestible oligosaccharides (14). Nevertheless, a cell model that can simulate the lamina propria environment and establish the allergic signal transduction through IEC, DC, and T cell is absent, which would be a powerful tool for food allergy investigations.

The Hippo pathway controls organ size by regulating cell growth, proliferation, and apoptosis, and participates in the development, oncogenesis, and immune responses (15, 16). When activated by upstream signals, the core kinases LATS1/2 are phosphorylated and activated, which further phosphorylate and inhibit the transcription factor YAP/TAZ, blocking the expression of downstream genes such as CTGF and Cyr61, finally suppressing cell proliferation (17). Previous studies have demonstrated the connection between the Hippo pathway and allergy (15), and the regulation of T cells by the Hippo pathway was also revealed (18, 19). Furthermore, Wang and colleagues found that the Hippo pathway involves in the allergenicity of shrimp allergen tropomyosin, provided potential targets for controlling food allergies (12).

Overall, this work tried to establish a three-dimensional (3D) intestinal cell model for food allergy investigations. To this aim, OVA was applied as a representative allergen, then DCs and T cells isolated from mouse spleen cells and the CMT93 cell line were used to establish the model. After optimization, the model was used to elucidate the connection between the Hippo pathway and food allergy. The results demonstrated that the 3D intestinal cell model was a powerful tool for food allergy investigations.

## 2 Materials and Methods

### 2.1 Reagents

LATS1 and Phospho-LATS1 (Thr1079) antibodies were from Cell Signaling Technology (MA, USA), TAZ antibody was from Santa Cruz Biotech (CA, USA), and β-actin antibody was from Boster Biological Technology (Wuhan, China). Flow cytometry antibodies (MHC-II-PerCP-Cyanine5.5, CD11c-FITC, CD4-FITC, IL-4-PE, and IFN-γ-APC) were from eBioscience (CA, USA). OVA was purchased from Sigma (MO, USA). Anti-Mouse CD4 Magnetic Particles-DM was purchased from BD Biosciences (NYC, USA).

### 2.2 Cell Culture

#### 2.2.1 CMT93 Cell Line

CMT93 cells were originally obtained from American Type Culture Collection (Virginia, USA). The cells were maintained in DMEM (KeyGENBioTECH, Nanjing, China) containing 10% fetal bovine serum and 1% penicillin-streptomycin solution (Sangon Biotech, Shanghai, China), and cultured at 37°C with 5% CO_2_.

#### 2.2.2 Spleen Cells

After sacrificed, the spleens of six-week-old female C57BL/6 mice were immediately obtained under aseptic conditions. Then the spleen was crushed and filtered through a 200 mesh sterile steel mesh, and the spleen cell suspension was collected (20).

#### 2.2.3 DC Separation

DCs were isolated from spleen cells according to the method of Cheng et al. with proper modifications (21). In brief, the spleen cell suspension was added to the cell layering agent with ρ=1.080, after centrifuging at 500 g for 5 min, the low-density cells were collected and resuspended in RPMI 1640 medium. After incubating for 3 h, the culture flask was shaken vigorously and the suspension was replaced by fresh RPMI 1640 medium. The operation was repeated 4 times, and the adherent cells were incubated overnight. Then the suspended cells were collected as isolated DCs. The purity of the DCs was analyzed by flow cytometry using MHC-II-PerCP-Cyanine5.5 and CD11c-FITC antibodies.

#### 2.2.4 T Cell Separation

The T cell isolation experiment is performed according to the manufacturer’s guidance of the magnetic bead sorting kit. In brief, spleen cells were labeled with BD IMag™ anti-mouse CD4 Particles-DM according to the magnetic labeling protocol. The labeled cell suspension was then placed within the magnetic field of the BD IMag™ Cell Separation Magnet. When the labeled cells migrated toward the magnet, the unlabeled cells in suspension were drawn off. The tube was then removed from the magnetic field for resuspension. The separation process was repeated twice to increase the purity of the cells.

### 2.3 Detection of Transepithelial Electrical Resistance (TEER) Value of CMT93 Cells

Trypsin-treated CMT93 cells were seeded on permeable transwell insert with 0.4 μm pore polyester membranes (Corning, USA) in a 12-well plate, after cultured at 37°C with 5% CO_2_ in DMEM (containing 10% fetal bovine serum and 1% penicillin-streptomycin solution) medium for the indicated time, and the resistance value was determined by a Millicell ERS-2 electrical resistance system (Millipore, USA). TEER was calculated according to the following formula:


$$\text{TEER}=(\text{Rs}－\text{R}0)\times 0.66{\text{ cm}}^{2}$$

Where Rs is the resistance value of the sample, and R0 is the resistance value of the blank (medium without cell).

### 2.4 Conventional Co-Culture System

0.5 mL of CMT93 cell culture medium with a density of 1×10^5^ cells/mL was seeded in each well of the 12-well plate and incubated for 36 hours. Then 0.5 mL 10^6^ cells/mL spleen cells were added, and OVA was added to each well as indicated 2 h later. The cells were further incubated for 36 h and then analyzed.

### 2.5 Transwell Co-Culture System

1×10^5^ CMT93 cells were seeded onto a transwell insert and placed into a 12-well plate. The cell culture medium was changed every 3 days until the cells were fully differentiated (TEER exceeding 220 Ω·cm2). Then 1 mL 5×10^5^ cells/mL spleen cells were added into the low-chamber. After incubating for 2 h, OVA was added to each well as indicated. The cells were further incubated for 36 h and then analyzed.

### 2.6 3D Intestinal Cell Model

1×10^5^ CMT93 cells were seeded onto a transwell insert and placed into a 12-well plate. The cell culture medium was changed every 3 days until the cells were fully differentiated (TEER exceeding 220 Ω·cm2). Then 1 mL 5×10^5^ cells/mL DCs were added into the low-chamber. After incubating for 2 h, OVA was added to each well as indicated. The cells were further incubated for 36 h, and the conditioned media in the low-chamber was centrifuged at 500 g for 5 min, the supernatant was added to a new well of 12-well plate with 5×10^5^ cells and 1 mL fresh medium. The cells were further incubated for 36 h and then analyzed.

### 2.7 Gene Knock-Down in CMT93 Cell

The sequence of the short hairpin RNA (shRNA) targeting TAZ mRNA was obtained from the MISSION^®^ shRNA database (Sigma-Aldrich, USA) and cloned into the lentiviral knock-down vector pLKO.1-Puro (Addgene, USA), and the empty pLKO.1-Puro vector was used for the negative control group. To prepare lentiviral particles, the vector was co-transfected with packaging plasmids (pLP1, pLP2, and pLP/VSVG) into HEK293T cells. Viral supernatants were harvested 48 h after transfection and applied to CMT93 with 10 μg/mL polybrene (Sigma-Aldrich, USA). 24 h later, the cells were incubated with 8 μg/mL puromycin to eliminate uninfected cells. After one week of selection, the knock-down efficiency was verified by qPCR, and the cells were ready for subsequential experiments. The primers for shRNA cloning are:

Forward (5’-3’)
CCGGCCAGGATTCAAGCACAGCTAACTCGAGTTAGCTGTGCTTGAATCCTGGTTTTTG
Reverse (5’-3’)
AATTCAAAAACCAGGATTCAAGCACAGCTAACTCGAGTTAGCTGTGCTTGAATCCTGG

### 2.8 Reverse-Transcription Quantitative PCR (qPCR)

Total RNA of cell samples was extracted using the E.Z.N.A.^®^ Total RNA Kit II (Omega Bio-Tek, USA). The reverse transcription of RNA to cDNA was performed using the HiScript^®^ II qRT Super kit (Vazyme Biotech, USA). The real-time quantitative PCR analysis was performed using the SYBR Green I kit (Vazyme Biotech, USA) with the LightCycler^®^ 480 II system (Roche, USA). The detailed procedure was as follows: initial denaturation at 95°C for 30 s, followed by 40 cycles of amplification (95°C for 10 s, 55°C for 30 s, and 72°C for 60 s), and data was collected at 72°C. The expression of GADPH was served as the internal standard for calibration. The sequences of qPCR primers are listed in **Table 1**.

**Table 1:** Primers for qPCR.

Genes	Forward (5’ – 3’)	Reverse (5’ – 3’)
*GATA3*	CTTATCAAGCCCAAGCGAAG	CCCATTAGCGTTCCTCCTC
*IL-4*	ACAGGAGAAGGGACGCCAT	GAAGCCCTACAGACGAGCTCA
*IFN-γ*	TGGCATAGATGTGGAAGAAAAGAG	TGCAGGATTTTCATGTCACCA
*T-bet*	TCAACCAGCACCAGACAGAG	AACATCCTGTAATGGCTTGTG
*OX40L*	CCCTCCAATCCAAAGACTCA	ATCCTTCGACCATCGTTCAG
*TSLP*	CAGCTTGTCTCCTGAAAATCG	AAATGTTTTGTCGGGGAGTG
*Il-33*	GACACATTGAGCATCCAAGG	AACAGATTGGTCATTGTATGTAC
*Il-25*	CTACAGACAGGCTCCCACATGGACC	CCTGCTGCTTCAGGTAGGGCTTTC
*GAPDH*	CACACCGACCTTCACCATTTT	GAGACAGCCGCATCTTCTTGT
*CTGF*	GGGCCTCTTCTGCGATTTC	ATCCAGGCAAGTGCATTGGTA
*Cyr61*	AGCGGGCAGTGCTGTGA	GTCGTCCAGGGAGTCCTTAATG
*TAZ*	GAGCAACATGGACGAGATGG	AGGTTAGAAAGGGCTCGCTT

### 2.9 Western Blot

The total protein from cells was extracted by Tissue Total Protein Lysis Buffer (Sangon Biotech, China), and the Western blot analysis was conducted with indicated antibodies as previously reported (12). In brief, 40 μg of total protein extract was denatured in loading buffer containing β-mercaptoethanol and SDS at 95°C and then loaded to each lane of a polyacrylamide gel (5% stacking gel and 12% separating gel). After being separated at 120 V, the proteins were transferred to a PVDF membrane. The membrane was then blocked by 5% BSA, incubated with indicated primary and secondary antibodies, and finally detected by ECL substrates with the FLUORChEM HD2 imaging system (PROTEINSIMPLE, USA).

### 2.10 Flow Cytometry

Cells were harvested after culture, and the intercellular markers (IL-4 and IFN-γ) were first stained with Transcription Factor Buffer plus relevant antibodies according to the manufacturer’s instructions. Then the surface markers were stained directly with 1 μg indicated antibody at room temperature for 30 minutes. The stained samples were analyzed by flow cytometry with a BECKMAN COULTER CytoFLEX, and the target cell subgroups were measured as designed using CytExpert 2.0 software.

### 2.11 Statistical Analysis

All the statistical results were analyzed with three replicates according to a completely randomized design. All data were performed by Analysis of variance (ANOVA). Data were analyzed statistically by repeated measures using SPSS 17.0 procedures and p < 0.05 was considered as statistically significant.

## 3 Results and Discussion

### 3.1 Optimizing Conventional Cell Models for Food Allergy Investigation

To establish a convenient food allergy cell model, we tried to combine different types of mouse cells to reconstitute the intestinal mucosal immune system. These cells were from the same species to guarantee intercellular communications. Moreover, compared with human-sourced cells, mouse-sourced cells are ideal materials, which are much easier to be obtained, especially for the primary cells.

The IECs and the immune cells in the lamina propria are the front-line against food allergens in the intestine. Therefore, we first tested the response of mouse IEC line CMT93 and immune cells from mouse spleen to common food allergens.

**Figure 1:**
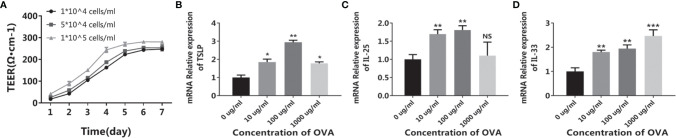
OVA activated the allergenic potential of CMT93 cells. CMT93 cells were cultured in a transwell dish for 7 days and then treated with different concentrations of OVA as indicated. The TEER value **(A)** and the transcription of TSLP **(B)**, IL-25 **(C)**, and IL-33 **(D)** were quantified. The results were represented as mean ± SD from three replicates. NS, not significant. *p < 0.05, **p < 0.01, ***p < 0.001.

After being cultured in a transwell insert for more than five days, the CMT93 cells tightly connected with each other, forming a compact monolayer cell with a relatively stable TEER around 25 Ω/cm, which simulated the intestinal epithelium and provided barrier function (**Figure 1A**). When treated with OVA, the expression of allergy-related cytokines of the monolayer was determined by qPCR. As shown in **Figures 1B**–**D**, TSLP, IL-25, and IL-33 were all upregulated after OVA stimulation. It has been reported that during food allergy or intestinal epithelium impartation, the IECs produce pro-allergenic cytokines including TSLP, IL-25, and IL-33, which triggered downstream cells such as DCs, Th2 cells, and innate lymphoid cells, finally induce food allergy (22, 23). Therefore, the above results indicated that the CMT93 monolayer is capable to simulate the allergenic responses of intestinal epithelium under OVA stimulation.

**Figure 2:**
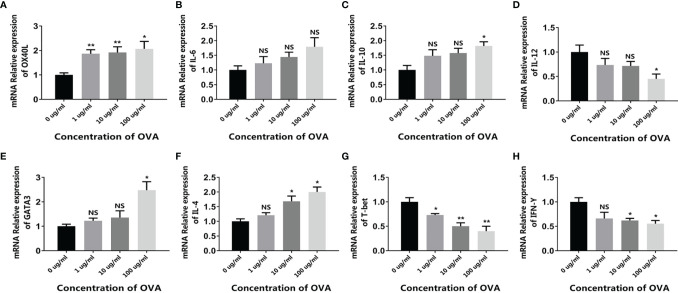
OVA activated the allergenic potential of spleen cells. Mouse spleen cells were treated by different concentrations of OVA as indicated. The transcription of OX40L **(A)**, IL-6 **(B)**, IL-10 **(C)**, IL-12 **(D)**, GATA3 **(E)**, IL-4 **(F)**, T-bet **(G)**, and IFN-γ **(H)** were determined by qPCR. The results were represented as mean ± SD from three replicates. NS, not significant. *p < 0.05, **p < 0.01.

When the intestinal mucosal immune system contacts with food allergens, the antigen-presenting cells such as DCs capture the antigen and present the antigen information to downstream T cells, initiating the allergen-specific responses. Based on this, we first isolated the immune cell mixture from the mouse spleen and tested the response to OVA *in vitro*. As shown in **Figure 2A**, when treated with OVA, the expression of DC surface protein OX40L significantly increased in an OVA-dose-dependent manner. Similarly, the cytokines IL-6 and IL-10 were also upregulated, whereas IL-12 was downregulated (**Figures 2B**–**D**). Furthermore, the expressions of T cell markers were also determined. Consistent with the DC results, the allergenic Th2 markers GATA3 and IL-4 were upregulated and the anti-allergenic Th1 markers T-bet and IFN-γ were downregulated (**Figures 2E**–**H**). It has been reported that allergenic DC expresses OX40L and IL-6 to communicate with T cells, leading to the expansion of Th2 cells. On the contrary, DC can also produce IL-12 to inhibit allergy, and IL-10 produced by DC or regulatory T cells can downregulate the whole immune responses and thus maintain immune homeostasis (24, 25). Consequently, these results demonstrated that OVA triggered the allergenic responses in the spleen cells, especially in the DCs and T cells.

Overall, these results indicated that OVA stimulated the allergenic response of IEC, DC, and T cells *in vitro*, implying the potential of these cells to be applied in establishing *in vitro* food allergy cell models.

### 3.2 Establishment of Co-Culture Food Allergy Cell Models

To reconstitute the crosstalk between IEC and immune cells, two co-culture systems containing CMT93 and mouse spleen cells were established.

**Figure 3:**
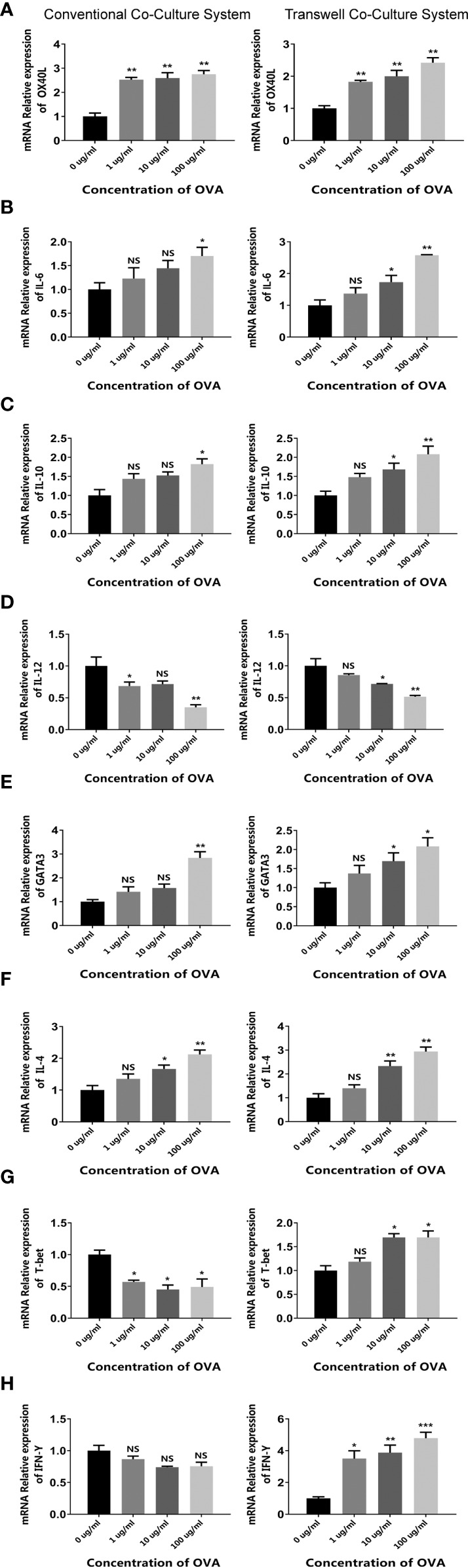
OVA activated the allergenic cell potential in the co-culture system. The conventional co-culture system (left panels) and the transwell co-culture system (right panels) were treated by different concentrations of OVA as indicated. The transcription of OX40L **(A)**, IL-6 **(B)**, IL-10 **(C)**, IL-12 **(D)**, GATA3 **(E)**, IL-4 **(F)**, T-bet **(G)**, and IFN-γ **(H)** were determined by qPCR. The results were represented as mean ± SD from three replicates. NS, not significant. *p < 0.05, **p < 0.01, ***p < 0.001.

For the first system, CMT93 and spleen cells were simply co-cultured in a single dish, forming a 2D co-culture model (conventional co-culture system). After being stimulated with OVA, the expression of allergenic DC and Th2 markers (including OX40L, IL-6, GATA3, and IL-4) and the regulatory cytokine IL-10 was upregulated, whereas the expression of anti-allergenic DC and Th1 markers (including IL-12, T-bet, and IFN-γ) was downregulated (**Figure 3**, left panels), which was consistent with the CMT93 and spleen cell culture models (**Figures 1** and **2**).

For the second system, CMT93 was seeded in a transwell insert and placed onto a 12-well plate with spleen cells, forming a transwell co-culture system that simulated the architecture of the intestinal mucosal immune system. When stimulated with OVA in the up-chamber, the DC and Th2 cell markers changed consistently with those in the conventional co-culture model and showed more significant dose-dependency with OVA. Interestingly, the Th1 markers were upregulated rather than downregulated in this model, implying different cell crosstalk in the two co-culture systems (**Figure 3**, right panels).

**Figure 4:**
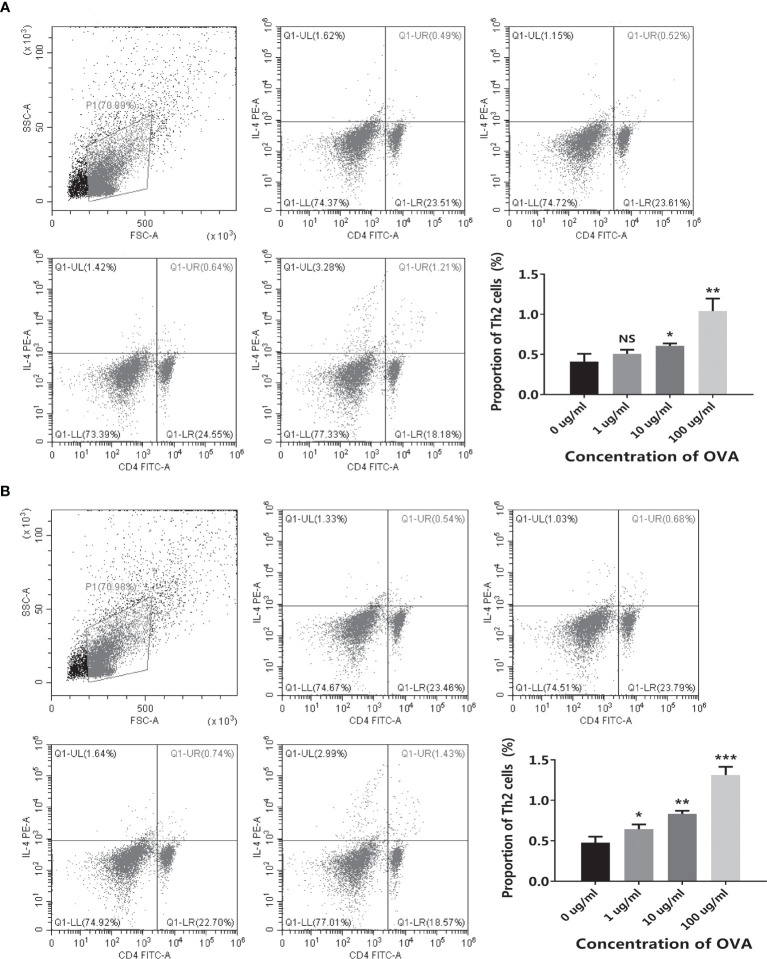
OVA stimulated Th2 cell differentiation in the co-culture system. The conventional co-culture system **(A)** and the transwell co-culture system **(B)** were treated by different concentrations of OVA as indicated. The proportion of CD4^+^IL-4^+^ Th2 cells was determined by flow cytometry. The representative cytometry plots and the statistical results were presented. The results were represented as mean ± SD from three replicates. NS, not significant. *p < 0.05, **p < 0.01, ***p < 0.001.

Moreover, the differentiation of T cells in OVA-stimulated co-culture systems was further assessed by flow cytometry. As shown in **Figure 4**, when stimulated by OVA, the proportion of Th2 was increased in both cell models. Noticeably, the transwell co-culture system (**Figure 4B**) showed more significant OVA-dose-dependency than the conventional co-culture system (**Figure 4A**).

In both systems, CMT93 and spleen cells can communicate with each other by producing cytokines. However, both CMT93 and spleen cells can directly interact with OVA in the conventional co-culture system but only CMT93 can directly interact with OVA in the transwell co-culture system. Therefore, the latter is more similar to the physiological environment, which might explain the better OVA-dose-dependency of this system.

### 3.3 Establishment of the 3D Intestinal Food Allergy Cell Model

**Figure 5:**
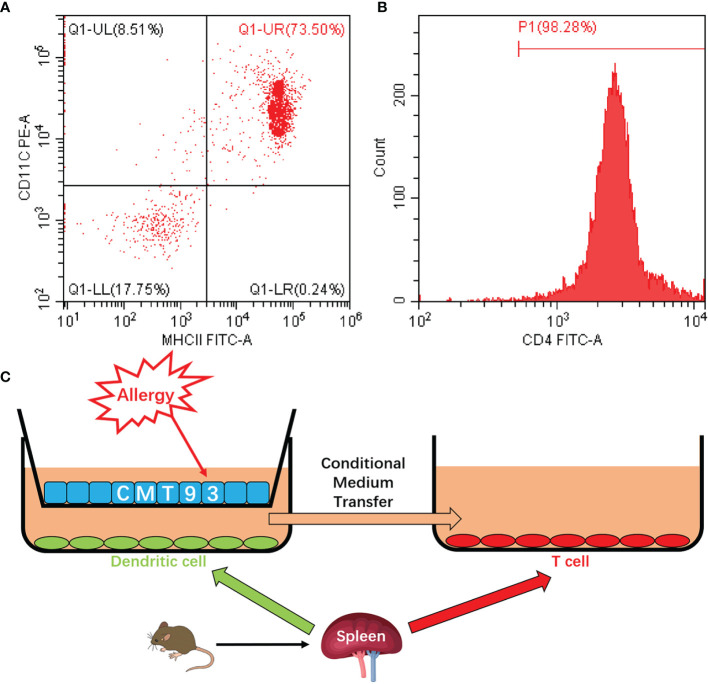
Establishment of the 3D intestinal cell model. **(A)** DCs were isolated from mouse spleen cells and the purity of CD11c^+^MHC-II^+^ cells was determined by flow cytometry. **(B)** T cells were isolated from mouse spleen cells and the purity of CD4^+^ cells was determined by flow cytometry. **(C)** Schematic diagram of the 3D intestinal cell model.

In the transwell co-culture system, the spleen cells are composed of various types of immune cells, such as DC, T cell, B cell, and macrophage, which made the system complex and impaired further applications. To establish a clearer system, the DCs and T cells were isolated from spleen cells. As shown in **Figures 5A, B**, flow cytometry results validated the high purity of the isolated cells. By using the purified DC and T cells, together with the CMT93 cell line, a clear cell model with the three types of cells was established (**Figure 5C**). CMT93 cells were cultured in a transwell insert and placed on a 12-well plate with DCs. After being stimulated by OVA, the conditioned media in the low-chamber, which contained the cytokines produced by DCs, was transferred to another culture dish with T cells. Then the allergy-related indexes in CMT93 cells, DCs, and T cells were assessed. This system, which we termed as 3D intestinal cell model, simulated the intestinal immune environment and separate different cell types, would be applied for food allergy investigations.

**Figure 6:**
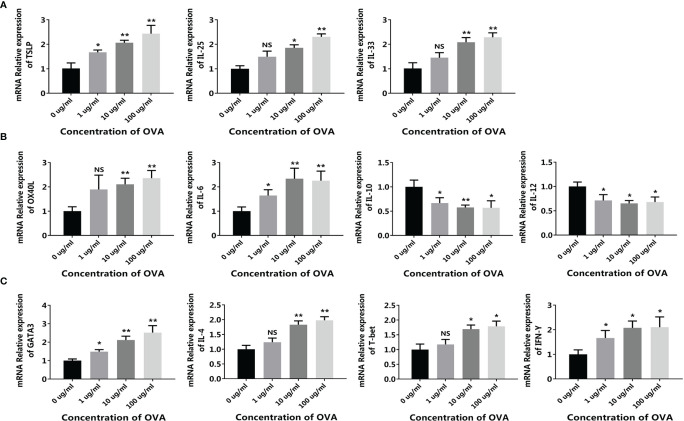
OVA activated the allergenic cell potential in the 3D intestinal cell model. The 3D intestinal cell model was treated by different concentrations of OVA as indicated. The transcription of TSLP, IL-25, and IL-33 in CMT93 cells **(A)**, OX40L, IL-6, IL-10, and IL-12 in DCs **(B)**, and GATA3, IL-4, T-bet, and IFN-γ in T cells **(C)** were determined by qPCR. The results were represented as mean ± SD from three replicates. NS, not significant. *p < 0.05, **p < 0.01.

Based on this model, different concentrations of OVA were added to the up-chamber and the change of allergy-related genes was measured in CMT93, DC, and T cells. As in the CMT93 cell culture model (**Figure 1**), OVA stimulated the expression of allergy-related genes including TSLP, IL-25, and IL-33 in CMT93 (**Figure 6A**). Noticeably, the gene expression in the 3D intestinal cell model showed better OVA-dose-dependence from 1 to 100 μg/mL.

The allergy-related genes in DC were also regulated by OVA (**Figure 6B**), similar to those in the conventional co-culture system and the transwell co-culture system (**Figure 3**), the expressions of OX40L and IL-6 were upregulated and the expression of IL-12 was downregulated. Interestingly, the expression of IL-10 was upregulated in the previous systems but downregulated in the 3D intestinal cell model, because IL-10 can be produced by DCs as well as other cell types such as Tregs, the 3D intestinal cell model avoid the disturbance of uncertain cells, and obtained clearer and more reliable results.

**Figure 7:**
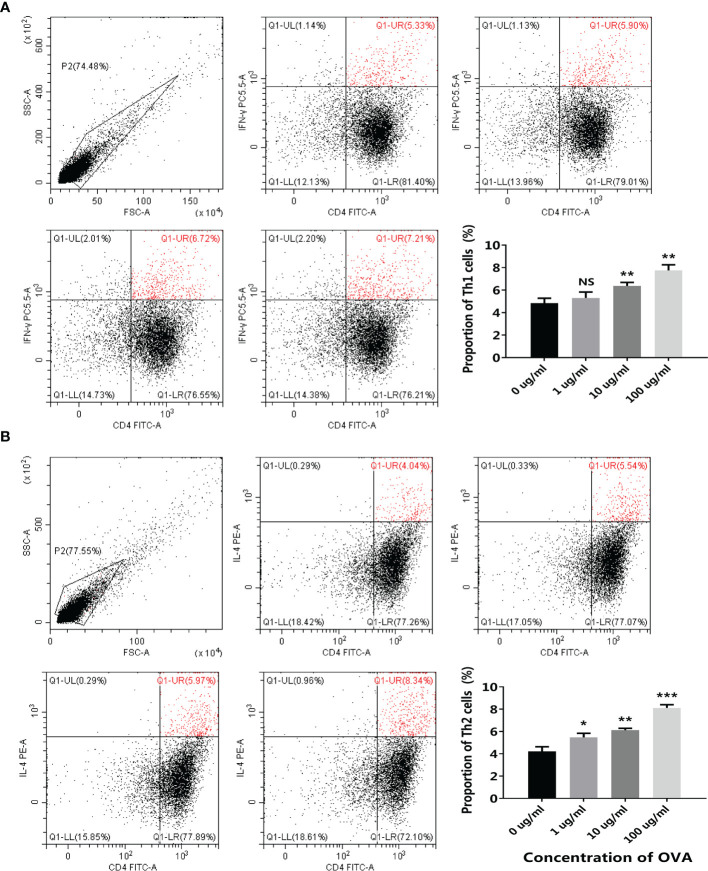
OVA stimulated T cell differentiation in the 3D intestinal cell model. 3D intestinal cell model was treated by different concentrations of OVA as indicated. The proportion of CD4^+^ IFN-γ^+^ Th1 cells **(A)** and CD4^+^IL-4^+^ Th2 cells **(B)** were determined by flow cytometry. The representative cytometry plots and the statistical results were presented. The results were represented as mean ± SD from three replicates. NS, not significant. *p < 0.05, **p < 0.01, ***p < 0.001.

After being stimulated by the conditioned media, the transcription of different T cell markers was determined by qPCR. As shown in **Figure 6C**, similar to the transwell co-culture system, both the Th1 markers (T-bet and IFN-γ) and the Th2 markers (GATA3 and IL-4) were upregulated after OVA stimulation. Flow cytometry further confirmed the results, as the proportion of both CD4^+^IFN-γ^+^ Th1 cells and CD4^+^IL-4^+^ Th2 cells were positively correlated with OVA concentration (**Figure 7**).

Overall, these results demonstrated that the 3D intestinal cell model can simulate the intestinal mucosal environment and reconstitute the main immune reactions during food allergy with the three types of cells, and therefore obtain more reliable data than the conventional cell systems.

### 3.4 Dissection of the 3D Intestinal Cell Model

**Figure 8:**
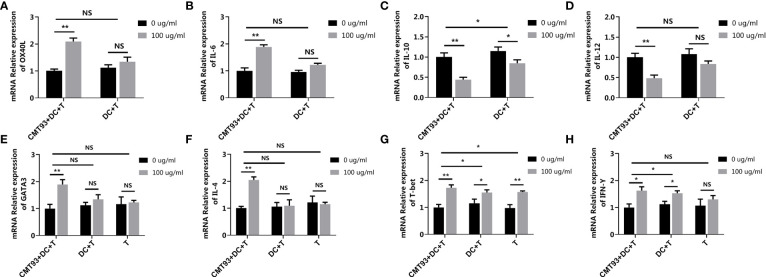
Dissection of the 3D intestinal cell model. The complete 3D intestinal cell model (CMT93+DC+T), the model lacking CMT93 cell (DC+T), and the model lacking CMT93 cell and DC (T) were treated by OVA as indicated. The transcription of OX40L **(A)**, IL-6 **(B)**, IL-10 **(C)**, IL-12 **(D)**, GATA3 **(E)**, IL-4 **(F)**, T-bet **(G)**, and IFN-γ **(H)** were determined by qPCR. The results were represented as mean ± SD from three replicates. NS, not significant. *p < 0.05, **p < 0.01.

To determine the specific role of each type of cell in the 3D intestinal cell model, the cell model was dissected to trace the allergenic signaling transduction. Based on the complete 3D intestinal cell model (CMT93+DC+T), CMT93 cells and DCs were removed successively, forming the DC+T cell model (DC+T) and the single T cell model (T). In the complete model, the expressions of OX40L and IL-6 were upregulated and the expressions of IL-10 and IL-12 were down-regulated after OVA stimulation in DCs. However, in the DC+T system lacking CMT93, the change of the expressions of OX40L, IL-6 and IL-12 were not significant, and the significance of IL-10 change was also reduced (**Figures 8A**–**D**).

Moreover, in the case of T cell-related genes, while OVA upregulated both Th1 and Th2 specific genes, lacking CMT93 cells and/or DCs eliminated the effect of OVA on Th2-, but not Th1-related genes (**Figures 8E**–**H**).

On the one hand, these results demonstrated that CMT93 cells, DCs, and T cells were all indispensable for the 3D intestinal cell model for food allergy investigation. On the other hand, the results demonstrated that Th2 rather than Th1 dominated OVA sensitization, which was consistent with previous studies and validated the reliability and specificity of the 3D intestinal cell model in food allergy investigations (7, 26).

### 3.5 Hippo Pathway in the Epithelial Cells Involved in OVA Sensitization

**Figure 9:**
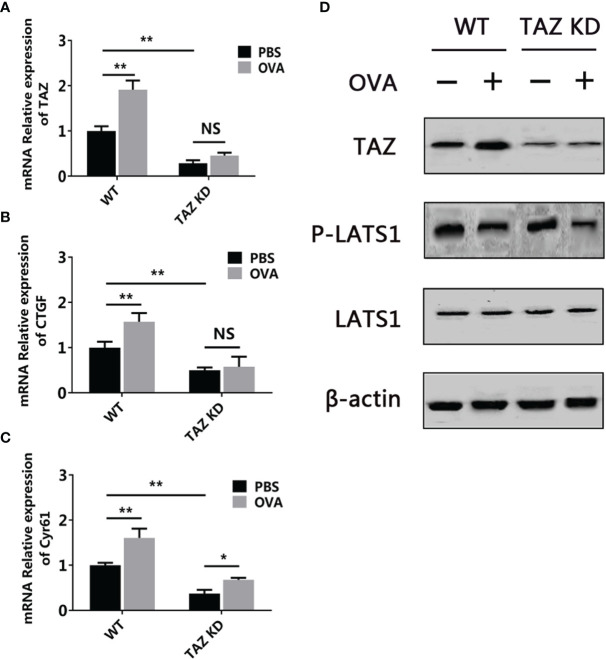
OVA inhibited the Hippo pathway in the 3D intestinal cell model. WT or TAZ knocked-down CMT93 cells were used to establish the 3D intestinal cell model. After being treated by OVA, the transcription of TAZ **(A)**, CTGF **(B)**, and Cyr61 **(C)** were determined by qPCR. **(D)** The protein level of TAZ, LATS1, phosphorylated LATS1 (P-LATS1), and β-actin (loading control) were determined by Western blot. The qPCR results were represented as mean ± SD from three replicates. NS, not significant. *p < 0.05, **p < 0.01.

A previous study has demonstrated the involvement of the Hippo pathway in food allergy using a mouse model (12). To further validate this finding and check the capability of the 3D intestinal cell model in food allergy research, the activation of the Hippo pathway was determined in this model. As shown in **Figure 9**, when stimulated by OVA, the Hippo pathway major transcription factor TAZ and downstream genes CTGF and Cyr61 were upregulated. Western blot further demonstrated that the protein level of TAZ was also increased and the phosphorylation of kinase LATS1 was decreased. All these results indicated that the Hippo pathway was inhibited during OVA sensitization. Moreover, when the TAZ gene was knocked-down, all these changes of the pathway were blocked, which validated the specificity of the phenomenon.

**Figure 10:**
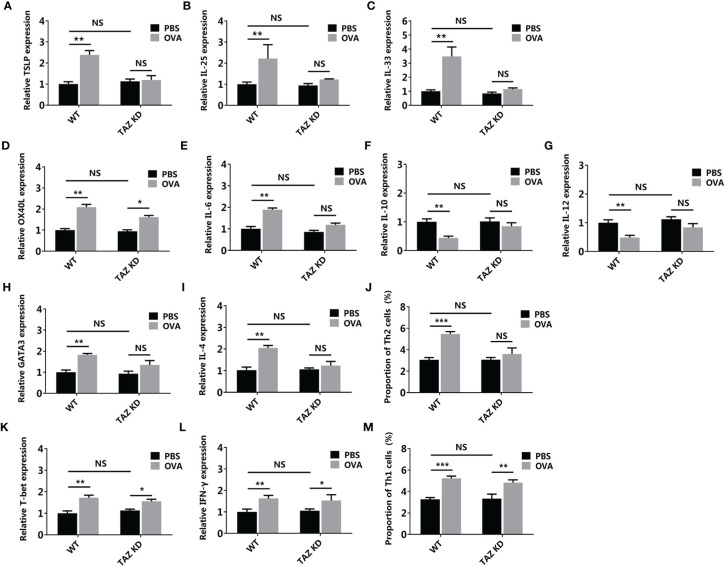
Hippo pathway in the epithelial cells involved in OVA sensitization. WT or TAZ knocked-down CMT93 cells were used to establish the 3D intestinal cell model. After being treated by OVA, the transcription of TSLP **(A)**, IL-25 **(B)**, IL-33 **(C)**, OX40L **(D)**, IL-6 **(E)**, IL-10 **(F)**, IL-12 **(G)**, GATA3 **(H)**, IL-4 **(I)**, T-bet **(K)**, and IFN-γ **(L)** were determined by qPCR. The proportions of Th2 **(J)** and Th1 **(M)** were determined by flow cytometry. The results were represented as mean ± SD from three replicates. NS, not significant. *p < 0.05, **p < 0.01, ***p < 0.001.

Based on the TAZ knocked-down CMT93, the 3D intestinal cell model was established (TAZ KD) (**Figure 10**). OVA failed to activate the allergenicity of CMT93 cell, DC, and Th2 cell in the TAZ KD group, as the expressions of TSLP, IL-25, IL-33, IL-6, IL-10, IL-12, GATA3, and IL-4, as well as the proportion of Th2 cells, were not significantly changed after OVA stimulation, and the expression change of OX40L was also less significant than in the wild-type (WT) system. On the contrary, OVA can still promote Th1 activation in the TAZ KD system, which included the upregulation of T-bet and IFN-γ expression, as well as the increased proportion of Th1.

Overall, by using the 3D intestinal cell model, we demonstrated that the Hippo pathway regulated Th2-involved OVA sensitization, which was consistent with the mouse model study, thus confirming the reliability of the 3D intestinal cell model in food allergy studies. Compared with the mouse models, this cell model depleted the disturbance of unrelated cells, making the results more solid and clearer. Gene manipulation is much more convenient in the cell model, facilitating the loss-of-function investigations.

## 4 Conclusion

In this study, a 3D intestinal cell model was established, which used three types of purified cells in a 3D architecture, which avoided unwanted interactions between cells and allergens but retained the essential communication for food allergy signal transduction. Compared with previous cell models, the 3D intestinal cell model showed more significant and more specific allergic responses, which were consistent with the results obtained from mouse models. Moreover, the 3D intestinal cell model is more convenient to be manipulated than the mouse models. Consequently, the 3D intestinal cell model is an ideal tool for food allergy investigations, especially for understanding the mechanisms of the sensitization stage, and would facilitate the studies in the field of intestinal mucosal immunity.

## Data Availability Statement

The raw data supporting the conclusions of this article will be made available by the authors, without undue reservation.

## Author Contributions

LF and YW designed research. CW analyzed data. WL and CW performed research. LF, WL, and CW wrote the paper. All authors contributed to the article and approved the submitted version.

## Funding

This study was financially supported by the National Key R&D Program of China (grant number 2019YFC1605002), and the National Natural Science Foundation of China (grant number 31871735).

## Conflict of Interest

The authors declare that the research was conducted in the absence of any commercial or financial relationships that could be construed as a potential conflict of interest.

## Publisher’s Note

All claims expressed in this article are solely those of the authors and do not necessarily represent those of their affiliated organizations, or those of the publisher, the editors and the reviewers. Any product that may be evaluated in this article, or claim that may be made by its manufacturer, is not guaranteed or endorsed by the publisher.
